# Mn^x+^ Substitution to Improve Na_3_V_2_(PO_4_)_2_F_3_-Based Electrodes for Sodium-Ion Battery Cathode

**DOI:** 10.3390/molecules28031409

**Published:** 2023-02-01

**Authors:** Renyuan Su, Weikai Zhu, Kang Liang, Peng Wei, Jianbin Li, Wenjun Liu, Yurong Ren

**Affiliations:** School of Materials Science and Engineering, Jiangsu Province Engineering Research Center of Intelligent Manufacturing Technology for the New Energy Vehicle Power Battery, Changzhou Key Laboratory of Intelligent Manufacturing and Advanced Technology for Power Battery, Changzhou University, Changzhou 213164, China

**Keywords:** sodium-ion battery, Na_3_V_2_(PO_4_)_2_F_3_, manganese ions doping, carbon coating

## Abstract

Na_3_V_2_(PO_4_)_2_F_3_ (NVPF) is an extremely promising sodium storage cathode material for sodium-ion batteries because of its stable structure, wide electrochemical window, and excellent electrochemical properties. Nevertheless, the low ionic and electronic conductivity resulting from the insulated PO_4_^3−^ structure limits its further development. In this work, the different valence states of Mn^x+^ ions (x = 2, 3, 4) doped NVPF were synthesized by the hydrothermal method. A series of tests and characterizations reveals that the doping of Mn ions (Mn^2+^, Mn^3+^, Mn^4+^) changes the crystal structure and also affects the residual carbon content, which further influences the electrochemical properties of NVPF-based materials. The sodiation/desodiation mechanism was also investigated. Among them, the as-prepared NVPF doped with Mn^2+^ delivers a high reversible discharge capacity (116.2 mAh g^−1^ at 0.2 C), and the capacity retention of 67.7% after 400 cycles at 1 C was obtained. Such excellent performance and facile modified methods will provide new design ideas for the development of secondary batteries.

## 1. Introduction

It is a big challenge to fulfill the rising demand for renewable energy with the current market mainstream lithium-ion batteries (LIBs) due to their high cost and low-capacity retention at high rates. Meanwhile, sodium-ion batteries (SIBs) occupy several advantages over LIBs, for instance, rich resources, wide distribution, and low prices [1,2,3,4,5]. Therefore, the SIBs were considered an extremely promising new energy-storage technology. However, the radius of Na^+^ (1.02 Å) is larger than that of Li^+^ (0.76 Å), which requires large sodium-ion transport channels, and inevitably causes volume change after long-term cycling [6]. More importantly, the breakthrough point for the electrochemical performance of the battery depends on the cathode materials. Therefore, considerable efforts have been put into finding suitable cathode materials for SIBs to solve these problems [7].

In recent times, transition metal oxides [8,9,10], polyanionic compounds [11,12], Prussian blue analogues [13,14], and organic compounds [15,16] make up the mainstream of cathode materials for SIBs [17]. Among them, Na_3_V_2_(PO_4_)_2_F_3_ (NVPF) and Na_3_V_2_(PO_4_)_3_ (NVP) with the NASICON type phosphates are considered to be the most favorable cathode materials owing to their three-dimensional open frameworks, which can boost sodium ion transport by delivering large interstitial spaces [18]. NVPF has also been demonstrated to possess a higher average working potential of ~3.9 V, and an ideal theoretical capacity of 128 mAh g^−1^, which is higher than that of NVP (~3.4 V, 117.6 mAh g^−1^) [17,19,20].

However, low electronic conductivity and sluggish Na^+^ diffusion limit the high rate performance and long-term cycling life of the NVPF material [21,22]. To improve the electrochemical performance, several methods are frequently used, heteroatom doping, particle nanosizing, and carbon coating [23,24]. Carbon coating is the most straightforward strategy to construct an external conductive framework to increase the surface electronic conductivity, while too much carbon coating prevents the electrolyte from being exposed to the electrode material entirely. Particle nanosizing can limit the material scale to nanometer, which can shorten the Na^+^ diffusion path and increase the exposure of electrodes to electrolytes at the same time. However, this will also lead to cracking at the electrode surface or particle agglomeration during cycling.

Heteroatom doping is a practical way to increase Na^+^ diffusion and structural stability. The common practice is using ions with similar radius size to replace sodium ions, which can increase the interplanar distance and facilitate the Na^+^ extraction/insertion [25,26]. However, limited by element species, fewer elements can be doped at sodium sites, and vanadium sites taking place is also widely studied (e.g., Mn^2+^, Zr^3+^, Fe^3+^, Ti^x+^). Some works found that transition metal elements such as Mn, Fe, and Ti are often used to modulate the width of the forbidden band. Moreover, transition metal ion doping has almost no effect on the material structure, but restricts the growth of microstructures in specific directions to obtain specific morphologies such as flakes and blocks, which generates more active sites at the edges of the electrode material to boost the intrinsic electronic conductivity [27,28,29].

Zhang et al. investigate the effect of Ti^x+^ (x = 2, 3, 4) doping on the NVPF compound properties. The appropriate amount of Ti^x+^ doping will inhibit the growth of NVPF crystals. At the same time, the particle size of the research object increases with the increase of the valence state, and the carbon content decreases with the increase of the valence state, resulting in the optimal cycle performance and rate performance of NVPF-Ti^2+^ [30]. Starting from the morphology design, Zhang et al. design a hollow microsphere structure of NVPF materials consisting of thin nanoplates and nanoparticles through a tetraglycol-assisted hydrothermal method, and then chemical vapor deposit is used to deposit a carbon layer on the surface of the designed NVPF to further optimize the electrochemical performance of the cathode materials with an initial reversible capacity of 122.9 mAh g^−1^ and a high coulombic efficiency of 99.1% even after 500 cycles at 5 C [31]. However, the effect of manganese ions with different valence states (Mn^x+^ x = 2, 3, 4) doping on the NVPF materials to modify the morphology by hydrothermal method and, accordingly, affect the electrochemical properties, has not been studied.

In this work, we report the synthesis of different valence states of manganese ions (Mn^2+^, Mn^3+^, Mn^4+^) doping in NVPF materials using a one-step hydrothermal method (hereafter referred to as NVM^2^PF@C, NVM^3^PF@C, NVM^4^PF@C). Moreover, the crystal structure, morphology, and carbon connect of the doped samples are also studied. Among them, NVM^2^PF@C exhibits an optimal initial discharge capacity of 116.2 mAh g^−1^ at 0.2 C and is maintained with a retention ratio of 67.7% even after 400 cycles at 1 C due to microcuboid morphologies and uniform carbon coating.

## 2. Results and Discussion

**Figure 1:**
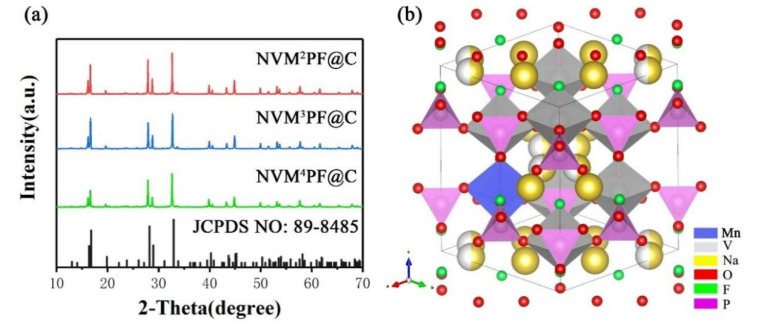
(**a**) XRD patterns of NVMPF@C; (**b**) crystal structure of NVMPF.

Figure 1a shows the XRD patterns of various NVPF samples. All diffraction peaks well correspond to tetragonal NVPF phases with a group of P4_2_/mnm (JCPDS NO: 89-8485) [24,32]. For these samples, no impurity peaks are detected, indicating that a low dose of Mn^x+^(x = 2, 3, 4) doping has no effect on the crystal structure of NVPF [33]. Moreover, no carbon-related diffraction peaks are observed, indicating that the presence of carbon may exist in the form of disordered carbon coating. In addition, embedding Mn ions can result in a larger cell volume since the radius of Mn ions (0.97 Å) is larger than that of V ions (0.78 Å). To verify this point, Jade software was used to study the influence of the doping of different valence states of manganese ions on the lattice parameters and cell volume (Table S1). It can be observed that all the samples doped with Mn ions show expansions both in cell parameters and volume, confirming that Mn ions were doped into the crystal lattice of NVPF successfully. Accordingly, the increased cell parameters and volume contribute to an increased Na^+^ migration rate and Na^+^ storage performance [34].

As shown in Figure 1b, the crystal structure of Na_3_V_2_(PO_4_)_2_F_3_ consists of bioctahedral units [V_2_O_8_F_3_] and tetrahedral units [PO_4_]. [V_2_O_8_F_3_] bioctahedrons are connected by two [VO_4_F_2_] octahedrons via F atoms, and the O atoms are all located on the [PO_4_] tetrahedrons. The Na(1) is located on a triangular prismatic site surrounded by two F atoms and four O atoms, and the other two extended triangular prismatic sites connected to the square prism of the F atoms are denoted as Na(2) sites [35,36]. Turning to the three sodium ions in NVPF, one pair of ions occupy the Na(1) site completely, while the remaining ion conquers half of the two Na(2) sites to make it a whole ion [37]. The vanadium ions with a smaller ionic radius are partially replaced by the Manganese ions with a larger ionic radius, broadening the transport of sodium ions and improving the transportation efficiency of sodium ions. At the same time, a low dose of Mn ions occupying the V sites shortens the bond length of V-O and P-O and lengthens the Na-O bond, which improves the intrinsic electronic conductivity [18].

**Figure 2:**
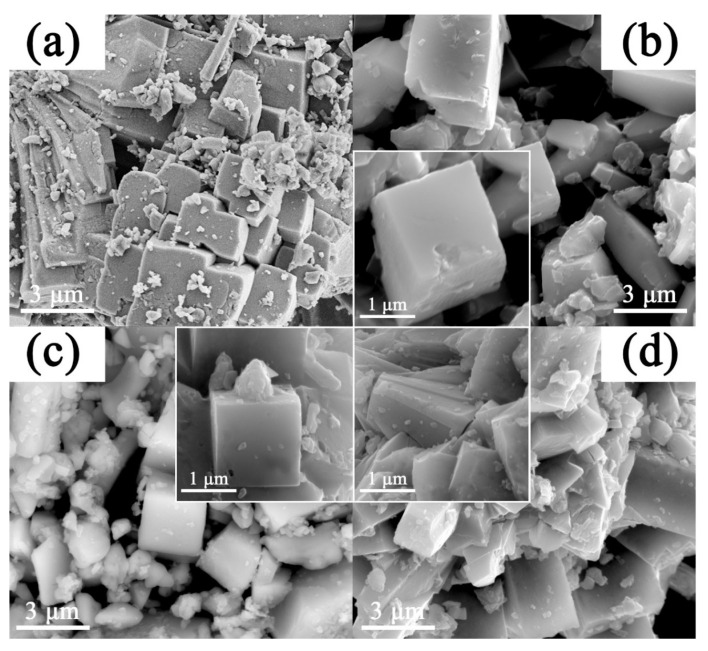
SEM patterns of (**a**) NVPF@C; (**b**) NVM^2^PF@C; (**c**) NVM^3^PF@C; (**d**) NVM^4^PF@C.

The effects of Mn^x+^(x = 2, 3, 4) doping on morphologies of the NVPF materials can be observed by SEM. Figure 2a depicts the morphology of the pristine NVPF@C. The overall structures are in blocky forms, and many broken particles are attached to the intact NVPF@C. From further high-magnification images in Figure 2b,c, it can be observed that both NVM^2^PF@C and NVM^3^PF@C materials show microcuboid morphologies with a length of about 2 μm. Compared with the pristine one, the modified materials are more uniformly dispersed and smaller in size, indicating that the introduction of Mn^x+^ (x = 2, 3) suppresses the crystal growth during a hydrothermal reaction. While in Figure 2d, the morphology of NVM^4^PF@C is close to that of the unmodified NVPF@C and even becomes more agglomerated and irregular.

**Figure 3:**
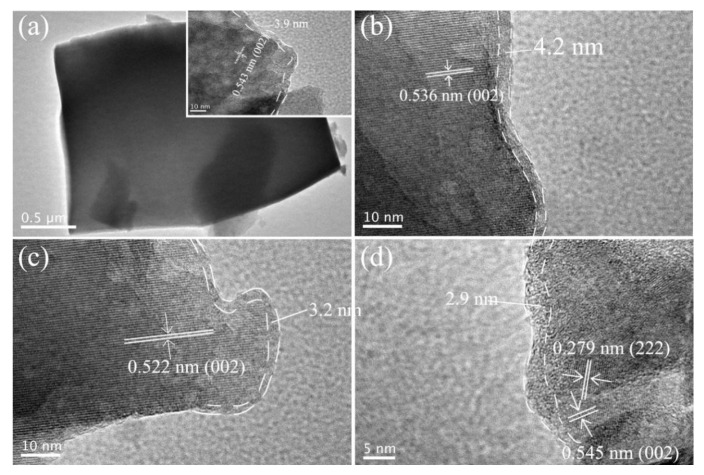
TEM images of (**a**) NVPF@C; (**b**) NVM^2^PF@C; (**c**) NVM^3^PF@C; (**d**) NVM^4^PF@C.

More information about lattice fringes and carbon coating could be obtained by TEM. As shown in Figure 3a, the pristine NVPF@C is in the form of block particles with a smooth surface, and its lattice planes with a spacing of 0.543 nm correspond to the (002) plane in tetragonal NVPF crystal. Simultaneously, the 3.9 nm thickness carbon coating can be observed. Turning to the NVM^2^PF@C, the Mn^2+^-doped NVMPF@C presents the same lattice planes (002) with the pristine NVPF@C material, and a thicker carbon layer (4.2 nm) is shown in Figure 3b. Similarly, Figure 3c presents the Mn^3+^-doped NVMPF@C sample. In particular, it can be seen that two lattice planes exist in the Mn^4+^-doped NVMPF@C with the spacing of 0.545 nm and 0.279 nm corresponding to the lattice plane (002) and (222), respectively, from Figure 3d. This phenomenon can be attributed to the strong oxide MnO_2_, which not only deteriorates the growth of NVPF crystals to induce growth in specific directions but also consumes a portion of citric acid leading to a 2.9 nm thickness carbon coating [38].

**Figure 4:**
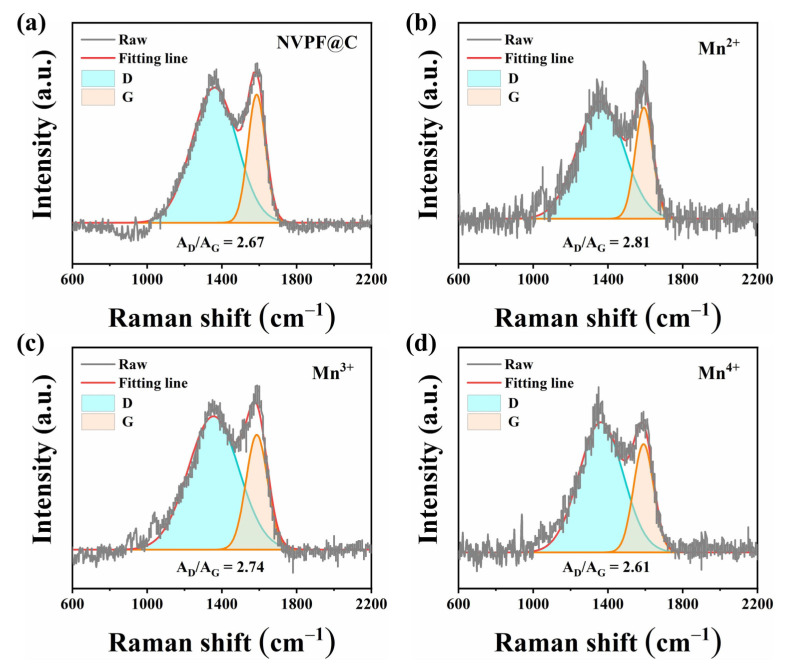
Raman images of (**a**) NVPF@C; (**b**) NVM^2^PF@C; (**c**) NVM^3^PF@C; (**d**) NVM^4^PF@C.

As shown in Figure 4, Raman spectra were characterized to investigate the carbon coating state. Two distinct peaks can be seen at 1350 cm^−1^ and 1600 cm^−1^ [39,40], corresponding to the disordered band (D-band) and graphite band (G-band), respectively. Furthermore, the D-band integral area to G-band integral area ratio (A_D_/A_G_) is used to describe the degree of graphitization. Figure 4 also illustrates the A_D_/A_G_ of the as-prepared NVM^x+^PF@C (x = 0, 2, 3, 4) samples, and the NVM^2+^PF@C possesses the highest A_D_/A_G_ value of 2.81, indicating that Mn^2+^-doped sample contains more electronic conductive disordered carbon [41].

The valence states of the series samples were examined using X-ray photoelectron spectroscopy (XPS) (Figure S1). From Figure S1a, the full XPS spectra show the characteristic peaks corresponding to the Na, V, P, O, and F elements, which verifies the NVPF material has been successfully synthesized. The high-resolution XPS spectra were used to analyze the Mn element of pristine and Mn^x+^ (x = 2, 3, 4) doped ones. Figure S1b,c show the F1s spectra of NVPF@C and NVM^2^PF@C. Compared with the pristine one, the divalent manganese ions doped sample possesses an extra Mn-F peak, which confirms the manganese ions were successfully doped into the pristine one [42]. Peaks at 653.8 eV and 645.9 eV in Figure S1d are associated with Mn^2+^ 2p_1/2_ and Mn^2+^ 2p_3/2_, respectively, both of which are characteristic peaks of Mn^2+^ 2p and only the peaks of Mn^2+^ can be seen in the NVM^2^PF@C sample [43]. Figure S1e shows the XPS spectrum of NVM^3^PF@C, excluding two characteristic peaks of Mn^2+^ 2p, the peak of Mn^3+^ locates at 641.8 eV [44]. In addition to the distinctive peaks of Mn^2+^ and Mn^3+^, Figure S1f also shows a typical peak caused by Mn^4+^ at 649.6 eV. The reason for this phenomenon is that citric acid has a reducing effect, and excess added citric acid can reduce Mn ions from a high valance state to a low valance state. Consequently, all of the valence states of manganese ions can be found in the NVM^4^PF@C sample. Similarly, both trivalent and divalent manganese ions are present in the NVM^3^PF@C sample. For NVM^2^PF@C, only the divalent valence state can be discovered, because the divalent is the lowest valence state of the manganese ion. Therefore, the XPS energy spectra of the manganese ion present multivalent states.

The carbon content of NVPF@C and NVMPF@C was evaluated by thermogravimetric analysis (TGA). As shown in Figure S2, the samples doped with different manganese ions possess different carbon content, and the value of NVPF@C, NVM^2^PF@C, NVM^3^PF@C and NVM^4^PF@C is 4.77%, 5.09%, 4.62%, and 4.23%, respectively (Table S2). At the same time, Figure S2 corroborates the presence of amorphous carbon coating and illustrates the slight difference in carbon content coincides with the thickness of carbon coating observed by TEM characterization. The XPS spectra also show that Mn^4+^ consumes the greatest amount of citric acid of all the samples, making the NVM^4^PF@C sample have the lowest carbon content (4.23%). The citric acid in the pristine and NVM^2^PF@C samples was used to reduce V^5+^ to V^3+^ and acted as a carbon source, so these two samples have a high carbon content of 4.77% and 5.09%, respectively. Turing to NVM^3^PF@C, whose carbon content (4.62%) is between that of NVM^2^PF@C and NVM^4^PF@C samples.

**Figure 5:**
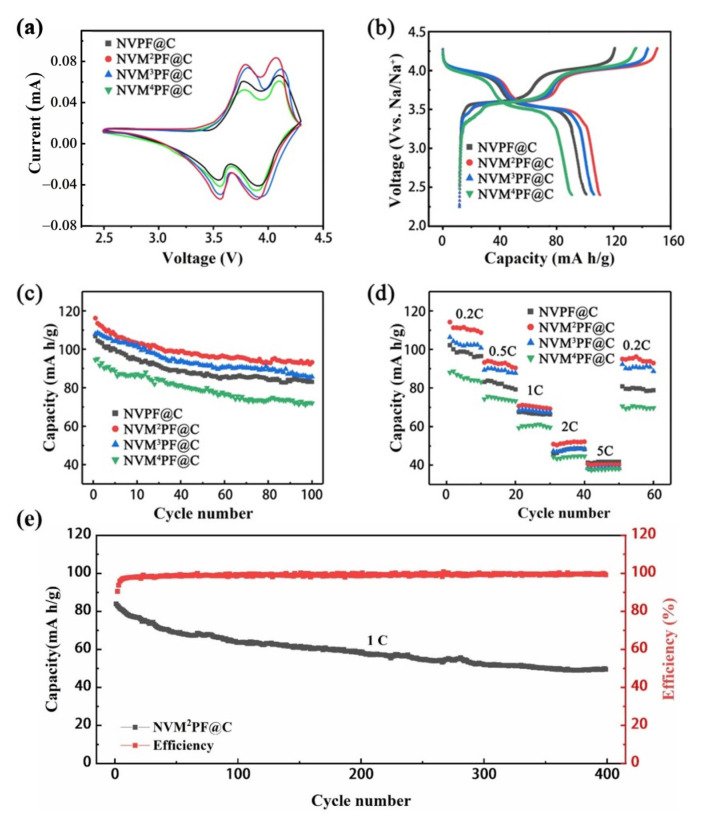
Electrochemical performances of NVPF@C and NVMPF@C electrode materials; (**a**) CV curves of different samples at 0.1 mV s^−1^; (**b**) charge-discharge curves of electrode materials; (**c**) cycling performance at 0.2 C; (**d**) rate capability of electrode materials; (**e**) long-cycling performance of NVM^2^PF@C at 1 C.

Figure 5a shows the cyclic voltammetry (CV) curves of Mn-doped NVPF@C and pristine NVPF@C materials measured in the voltage range of 2.5 to 4.3 V (vs. Na^+^/Na) at 0.1 mV s^−1^. The redox peak positions for the NVM^2^PF@C electrode are at 3.596/3.758 V and 3.867/4.102 V, which is assigned to the V^3+^/V^4+^ redox couple [40], as can be observed from the CV curves. NVM^2^PF@C has a cathodic potential difference of 162 mV and an anodic potential difference of 235 mV, which are 59 mV and 10 mV, respectively, less than the pristine NVPF@C electrode. Additional details regarding the cathodic and anodic potential differences between various samples were calculated, which is shown in Table S3. By comparison, the NVM^2^PF@C electrode has the fewest cathodic and anodic potential differences, indicating a decreased polarization and enhanced Na^+^ diffusion kinetics [45,46,47].

Figure 5b shows the initial charge/discharge curves of NVPF@C, NVM^2^PF@C, NVM^3^PF@C, and NVM^4^PF@C at 0.2 C. The pristine electrodes possess two obvious charge/discharge plateaus, 3.57/3.79 V and 3.83/4.07 V. While, the charge/discharge plateaus of NVM^2^PF@C corresponding to 3.60/3.76 V and 3.87/4.10V, which has a smaller polarization compared with the NVPF@C. The result is consistent with the obtained data from the CV plots and table. In addition, the divalent manganese ion doping electrode has the best initial discharge capacity of all the electrodes, as shown by the longer charge/discharge plateau of NVM^2^PF@C than other samples, which is 116.2 mAh g^−1^ at 0.2 C, close to the theoretical value (128 mAh g^−1^). While, the values of the other three ones are 106.8 mAh g^−1^, 108.1 mAh g^−1^, and 94.5 mAh g^−1^ corresponding to NVPF@C, NVM^3^PF@C, and NVM^4^PF@C, respectively. Furthermore, the increased initial specific capacity is benefiting from smaller particles, which can broaden the region in contact between the electrode and electrolyte, thereby enhancing sodium ion transport [48,49].

The cycling performance of the as-prepared objects is depicted in Figure 5c (NVPF@C, NVM^2^PF@C, NVM^3^PF@C, NVM^4^PF@C). The NVM^2^PF@C electrode shows the highest initial discharge capacity of 116.2 mAh g^−1^ and remains 93.2 mAh g^−1^ at 0.2 C after 100 cycles. The capacity retention is 80.2%. While, the NVPF@C, NVM^3^PF@C, and NVM^4^PF@C electrodes display the initial discharge capacity of 106.8 mAh g^−1^, 108.1 mAh g^−1^, and 94.5 mAh g^−1^ after cycling, corresponding to 77.8%, 79.3%, and 73.9% capacity retention, respectively (Table S4). Benefiting from the small particles with micro-sized distribution, the NVM^2^PF@C and NVM^3^PF@C samples possess a favorable cycling performance. The manganese ion doped samples induce zero-dimensional defects that can lead to more active sites, larger sodium-ion transport channels, and a more stable crystal structure [50]. However, the tetravalent manganese ions doped sample shows an inferior result because of low carbon content and agglomerated and broken particles, deteriorating the electrochemical performance in contrast with the NVPF@C material.

Figure 5d displays the rate performance of the four electrodes. At the current rate of 0.2, 0.5, 1, 2, and 5 C, the capacity ranking presented by rate curves among the four samples is in line with the cycling performance at 0.2 C (from first to fourth is NVM^2^PF@C, NVM^3^PF@C, NVPF@C, and NVM^4^PF@C). The data reveal that both the NVPF@C materials doped with divalent and trivalent manganese ions present high rate performance at different current rates. The reason behind this phenomenon could be related to the enhanced structural stability carried on by the partial replacement of manganese ions for vanadium sites. In particular, the NVM^2^PF@C electrode possesses the optimal capacity compared with the other three samples. Classical chemical structure theory states that structure determines performance, during a high current rate, the structural stability is expressed as cycling stability. When the rate was shifted from 5 C to 0.2 C, the capacities of NVM^2^PF@C recovered to 99.3 mAh g^−1^, revealing an outstanding electrochemical reversibility. The long-life cycling performance of the NVM^2^PF@C electrode is provided in Figure 5e. The discharge capacity reaches 83.9 mAh g^−1^ and a retention ratio of 67.7% after 400 cycles at 1 C, indicating that the divalent manganese ions replacing part of the vanadium ions can deliver better long-term cycling life.

The XRD pattern and the SEM images were used to study the phase analysis and morphology changes of the NVM^2^PF@C cathode before and after 400 cycles at 1 C. The diffraction peak positions of the NVM^2^PF@C all match well with the Aman space group (JCPDS NO: 89-8485), and no impurity peaks can be observed in Figure S3. It can be concluded that the NVM^2^PF@C electrode is still maintained in the NVPF phase, which proves that divalent manganese ions doping is beneficial to structural stability and cycling stability [51]. However, the diffraction peak positions of the NVM^2^PF@C sample after cycling shift to lower angles, indicating that the cell parameters and the lattice spacing get increased. This probably results from the sodium-ion transport channels that have been further expanded after long cycling, which makes the crystal structure larger. Simultaneously, the relative intensity of the diffraction peaks after cycling is weaker than that before cycling, demonstrating a slightly decreased crystallinity. This phenomenon is mainly associated with the deteriorated crystal structure, and the other possible reason is the coating of mixed slurry (conductive agent and binder). The NVM^2^PF@C sample exhibits a uniform cubic morphology when comparing the morphology before and after cycling, despite the fact that a lot of broken particles have adhered to the surface of the long-term cycling electrode. This outcome is consistent with the favorable reversible cycling performance and the electrochemical performance analysis discussed above.

**Figure 6:**
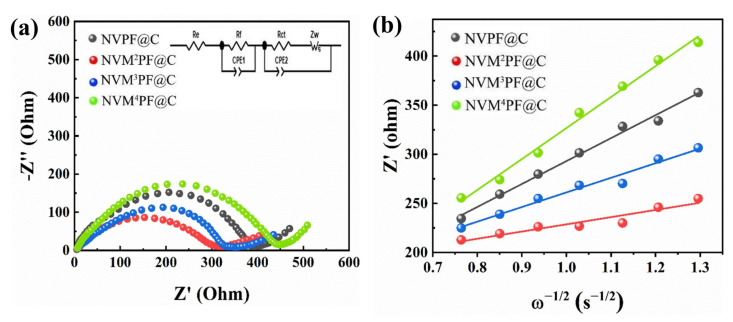
(**a**) Nyquist plots; (**b**) the relationship between Zre and ꞷ^−1/2^ in the low-frequency region of different samples charged to 4.1 V.

Electrochemical impedance spectroscopy (EIS) was used to future investigate the four samples’ electrochemical behavior and kinetic reaction process. The Nyquist plots, as illustrated in Figure 6a, consist of a semicircle at high frequency and a sloping line at low frequency. The semicircle at high frequency is associated with the diffusive migration of sodium ions via the solid electrolyte interface (SEI), and the sloping line at low frequency is connected with the solid diffusion process of sodium ions inside the active material particles. In the inset figure, the equivalent circuit is shown with *R_e_*, *R_ct_*, *R_f_*, *CPE_1_, CPE_2_,* and *Zw*, which stand for, electrolyte resistance, interfacial charge transfer resistance, film resistance of Na^+^ diffusion, capacitance of the passivation film, double layer and Warburg impedance, respectively [52]. The *R_ct_* of the NVM^2^PF@C electrode is 82.5 Ω (Table S5), which is much lower than that of the other three (NVPF@C 110.0 Ω NVM^3^PF@C 91.7 Ω NVM^4^PF@C 136.9 Ω), implying the Mn^2+^-doped NVPF@C possesses the enhanced Na^+^ transfer kinetic and higher electronic conductivity. The smaller the radius of the semicircle, the less the sloping line’s angle of inclination is less than 45°, which reflects the roughness of the electrode surface. It seems that Mn-doped samples’ particle surface roughness and specific surface area are increased.

The coefficient diffusion of sodium ion (*D_Na+_*) could be obtained in view of Equation (1) [30]:

(1) $$D=\frac{R^{2}T^{2}}{{2A}^{2}n^{4}F^{4}C^{2}\sigma^{2}}\;$$

The following Equation (2) can be used to compute the Warburg factor (*σ*) linked to *Z’*. Where *R* is the gas constant, *T* is the absolute temperature, *A* is the surface area, *n* is the number of electrons transferred per molecule, *F* is the Faraday constant, and *C* is the concentration of Na^+^

(2) $$Z_{\mathrm{re}}{=R}_{e}{+R}_{\mathrm{ct}}{+\sigma \omega }^{-1/2}$$

where *ꞷ* stands for the angle frequency. The Warburg factor can be observed in Figure 6b, which reveals the relationship between the linearly fitting *Zre* and *ω*^−1/2^ in the low frequency, and the calculated results for the series of NVM^x+^PF@C are displayed in Table S5. The NVM^2^PF@C electrode possesses the highest sodium-ion diffusion coefficient reaching 1.03 × 10^−13^ cm^2^ s^−1^. While the values of the others are much lower than that of the NVM^2^PF@C. The uniform and porous grain morphology after a proper Mn^2+^ concentration doping effectively reduces interfacial resistance and improves the Na^+^ diffusion kinetics. At the same time, a low dose of the divalent manganese ion doping helps to stabilize the Na^+^ diffusion channel and optimize the NASICON structure [37]. Furthermore, the uniform carbon coating plays a very important part in the charge/discharge process, which significantly increases the intrinsic electronic conductivity. Therefore, the NVM^2^PF@C and NVM^3^PF@C electrodes have improvements in both reversible capacity and cycling performance compared with the pristine NVPF@C electrode.

## 3. Experimental

The NVPF@C was prepared by a hydrothermal method. The components are NaF (Macklin 99.5%), NH_4_H_2_PO_4_ (Macklin 99.5%), NH_4_VO_3_ (Macklin, 99.5%), C_6_H_8_O_7_ (Macklin, Ar), and manganese source including (CH_3_COO)_2_Mn, (CH_3_COO)_3_Mn•2H_2_O and MnO_2_(Macklin, Ar) in the molar ratio of 3:2:1.95:1.5:0.05. Firstly, NH_4_VO_3_ and C_6_H_8_O_7_ were dissolved in an appropriate amount of deionized water, stirring at 70 °C to obtain a transparent solution. Then, NH_4_H_2_PO_4_, NaF, and manganese sources were put into the above solution. The color of the solution changed from faint yellow to brick red after 30 min of stirring. Next, a 100 mL Teflon-lined autoclave was filled with the previously prepared solution and heated at 200 °C for 12 h. After that, until the as-prepared samples returned to 25 °C, the dark green product was obtained then centrifuged and washed to acquire the precursor. The above precursor was put into a tube furnace for calcination in Ar/H_2_. The first step was preheated at 300 °C for 4 h, and the second step was sintered to 600 °C for 8 h. Then, the black powder product Na_3_V_1.95_Mn_0.05_(PO_4_)_2_F_3_@C (referred to as NVMPF@C) was obtained. According to the different valence states of manganese sources (Mn^2+^, Mn^3+^, Mn^4+^), a series of NVMPF@C materials (NVM^2^PF@C, NVM^3^PF@C, NVM^4^PF@C) can be obtained.

Phase composite and crystal structure of all NVM^x+^PF@C (x = 0, 2, 3, 4) samples were performed via X-ray diffraction (XRD). Jade software was used to study the lattice parameters. X-ray photoelectron spectroscopy (XPS) analysis was identified by a Thermo VG Scientific ESCALab 250. The Thermogravimetric (TG) data of the series of NVPF@C samples were tested by Labsys Evo/seteram thermal analyzer. Raman spectra were obtained by using a Raman spectrometer (LabRAM-HR) with an excitation wavelength of 532 nm. High Resolution field transmission electron microscopy (FETEM, a JEOL 2100) and field emission scanning electron microscopy (FESEM, SUPRA55) were employed to explore the surface morphology of the NVM^x+^PF@C and electrodes.

The active substance, conductive agent (Super p), and binder (PVDF) were weighed at a mass ratio of 7:2:1 and thoroughly ground in a mortar. An appropriate amount of NMP solvent was added drop by drop and mixed evenly. The above slurry was coated on aluminum foil and put into a vacuum oven to evaporate solvent and water. After 12 h, the as-prepared electrode was cut into disks. In our experiment, the average mass loading of the active materials was 1.2–1.5 mg cm^−2^ on a 12 mm disk. The batteries were assembled in the argon-filled glove box (BRAUN). A proper amount of electrolyte (1 M NaClO_4_ in EC:DMC = 1:1 with 5%FEC) was added to obtain sodium ion half-cell coin cells (CR2032 type). Electrochemical performance measurements were evaluated by a battery test system (LAND), and the potential range was set from 2.5 V to 4.3 V. Cyclic voltammogram (CV, 2.5–4.3 V) was tested by an electrochemical workstation (CHI 600A) with a sweep rate of 0.1 mV s^−1^. Electrochemical impedance spectroscopy (EIS) was investigated by an electrochemical workstation (CHI 600A), and the frequency ranged from 0.1 MHz to 10^5^ MHz.

## 4. Conclusions

In summary, by using a single hydrothermal method, manganese ions with different valence states doped NVPF@C were successfully synthesized. The manganese with different valence states was used to investigate the effects of NVPF, such as morphologies, the thickness of carbon coating layer, and electrochemical performance. It was found that the Mn^2+^ can effectively guide the formation of microcuboid NVPF and restrict the size growth of NVPF crystal. In addition, the Mn^2+^ doping strategy also improved the electrochemical performance by lower Rct and larger Na^+^ diffusion kinetics. Thus, the NVM^2^PF@C sample performed the best cycling and rate performance with the initial discharge capacity of 116.2 mAh g^−1^ and the capacity retention (67.7% after 400 cycles at 1 C). The improved electrochemical performance is attributed to the uniform carbon coating and stable crystal structure, indicating that divalent manganese ions doping NVPF@C can be a promising cathode for SIBs.

## Data Availability

Not applicable.

## Supplementary Materials

The following supporting information can be downloaded at: https://www.mdpi.com/article/10.3390/molecules28031409/s1, Figure S1: Full XPS spectrum of NVPF@C; high-resolution XPS spectra of NVPF@C and NVMPF@C; Figure S2: The TG curves of NVPF@C and NVMPF@C; Figure S3: XRD and SEM images of NVM^2^PF@C before and after the cycles; Table S1: Cell parameters of NVPF@C and NVMPF@C; Table S2: Carbon content of NVPF@C and NVMPF@C; Table S3: Potential difference of NVPF@C and NVMPF@C; Table S4: Long-term cycling performance of NVPF@C and NVMPF@C; Table S5: The impedance parameters from the simulated equivalent circuit and sodium diffusion coefficients of NVPF@C and NVMPF@C.

## Sample Availability

Samples of the compounds are not available from the authors.
