# Supplementary material for: Mnx+ Substitution to Improve Na3V2(PO4)2F3-Based Electrodes for Sodium-Ion Battery Cathode
Source: Molecules. 2023 Feb 1;28(3):1409. doi: 10.3390/molecules28031409 (PMC9920057; doi:10.3390/molecules28031409)
Supplement: Supplementary file 1 [file molecules-28-01409-s001.zip › molecules-2166208-supplementary.pdf]

Supporting information

Mn^{x+} Substitution to Improve $Na_3V_2(PO_4)_2F_3$-Based Electrodes for Sodium-Ion Battery Cathode

Renyuan Su, Weikai Zhu, Kang Liang, Peng Wei *, Jianbin Li, Wenjun Liu and Yurong Ren *

School of Materials Science and Engineering, Jiangsu Province Engineering Research Center of Intelligent Manufacturing Technology for the New Energy Vehicle Power Battery, Changzhou Key Laboratory of Intelligent Manufacturing and Advanced Technology for Power Battery, Changzhou University, Changzhou 213164, China; surenyuan1021@163.com (R.S.); 18862489151@163.com (W.Z.); isliangkang@163.com (K.L.); jianbinchem@cczuedu.cn (J.L.); 00003203@cczu.edu.cn (W.L.)

* Correspondence: weipeng@cczu.edu.cn (P.W.); ryrchem@cczu.edu.cn (Y.R.)

Figures and Tables

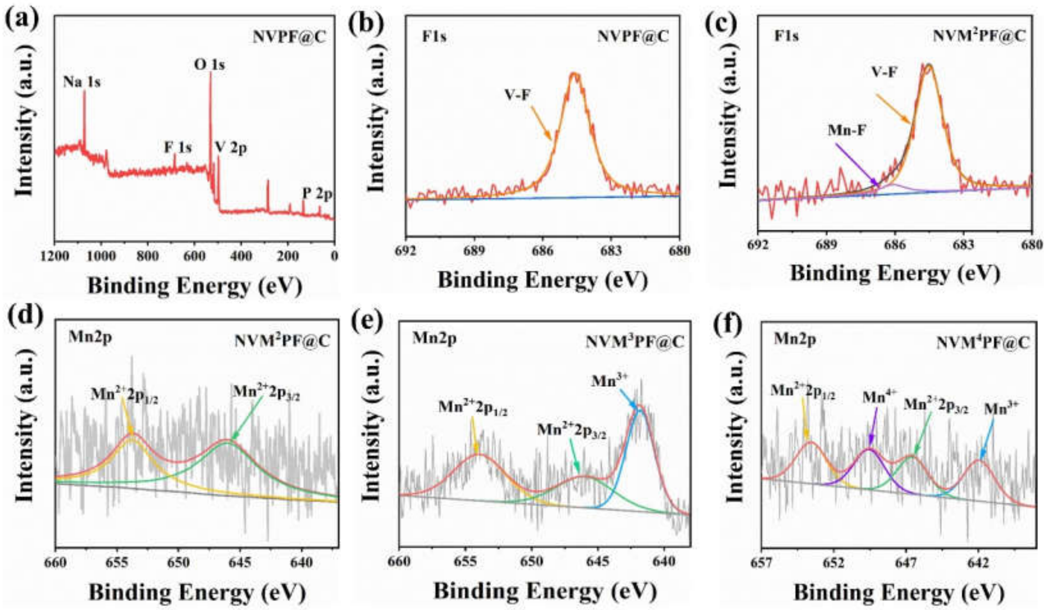

Figure S1. (a) Full XPS spectrum of NVPF@C; (b) high-resolution XPS spectra for F1s of NVPF@C; (c)high-resolution XPS spectra for F1s of $NVM^2PF@C$; (d) high-resolution XPS spectra for Mn2p of $NVM^2PF@C$; (e)high resolution XPS spectra for Mn2p of $NVM^3PF@C$(a) (f) high-resolution XPS spectra for Mn2p of $NVM^4PF@C$

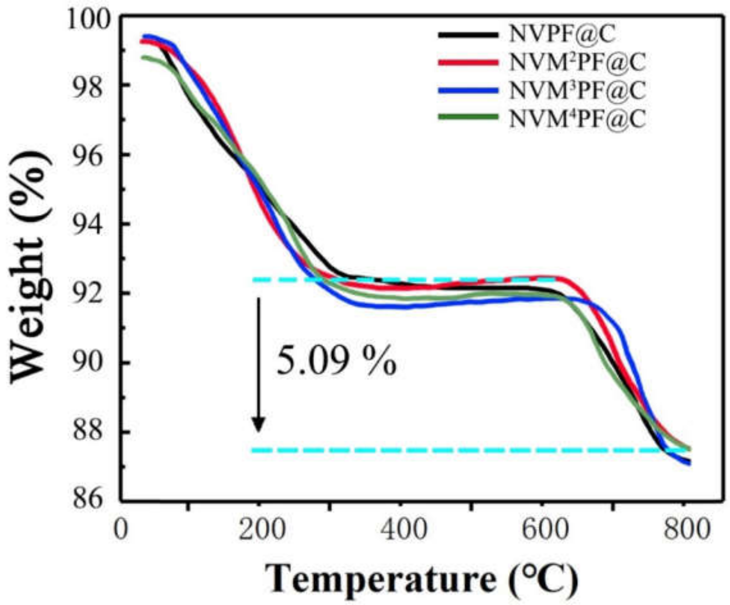

Figure S2. The TG curves of NVPF@C and NVMPF@C

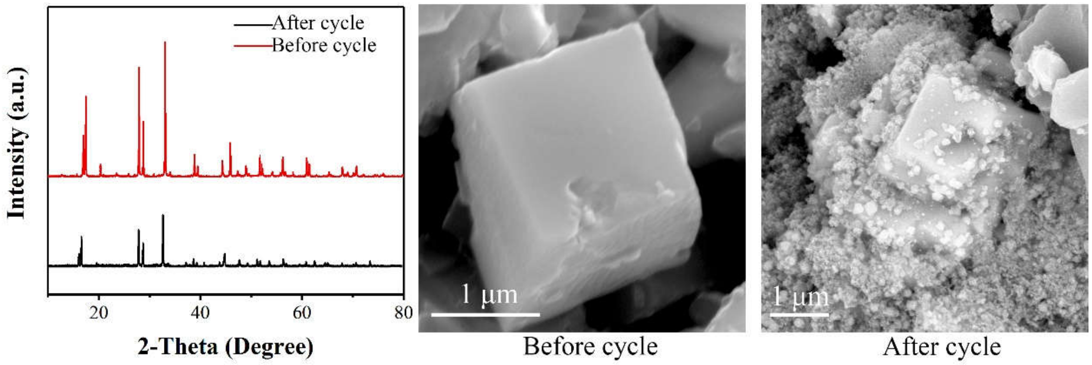

Figure S3. XRD and SEM images of $NVM^2PF@C$ before and after the cycles

Table S1. Cell parameters of NVPF@C and NVMPF@C

Samples	a(Å)	b(Å)	c(Å)	V(Å^3)
NVPF	9.047	9.047	10.705	876.185
NVM^2PF	9.093	9.093	10.861	898.016
NVM^3PF	9.085	9.085	10.799	891.319
NVM^4PF	9.064	9.064	10.735	881.946

Table S2. Carbon content of NVPF@C and NVMPF@C

Samples	Carbon content %
NVPF@C	4.77%
$NVM^2PF@C$	5.09%
$NVM^3PF@C$	4.63%
$NVM^4PF@C$	4.23%

Table S3. Potential difference of NVPF@C and NVMPF@C

Samples	Potential difference (mv)	
NVPF@C	221	245
$NVM^2PF@C$	162	235
$NVM^3PF@C$	228	203
$NVM^4PF@C$	214	299

Table S4. Long-term cycling performance of NVPF@C and NVMPF@C

Samples	1 st cycle Discharge capacity (mAh g^{-1})	100 th cycle Discharge capacity (mAh g^{-1})	Retention (100%)
NVPF@C	106.8	83.1	77.8
$NVM^2PF@C$	116.2	93.2	80.2
$NVM^3PF@C$	108.1	85.7	79.3
$NVM^4PF@C$	94.5	69.9	73.9

Table S5. The impedance parameters from the simulated equivalent circuit and sodium diffusion coefficients of NVPF@C and NVMPF@C

Samples	R_f (Ω)	R_{ct} (Ω)	σ (Ω cm^2 $s^{-1/2}$)	D_{Na^+} (cm^2 s^{-1})
NVPF@C	395	110.0	231.9	6.61×10^{-14}
$NVM^2PF@C$	317	82.5	74.2	1.03×10^{-13}
$NVM^3PF@C$	345	91.7	157.7	8.23×10^{-14}
$NVM^4PF@C$	441	136.9	346.9	3.27×10^{-14}
